# The Therapeutic Potential of Gut-Microbiota-Derived Metabolite 4-Phenylbutyric Acid in *Escherichia coli*-Induced Colitis

**DOI:** 10.3390/ijms26051974

**Published:** 2025-02-25

**Authors:** Kui Wang, Yuan Hu, Yu Wu, Jie Xu, Yiyi Zhao, Jing Yang, Xiaobing Li

**Affiliations:** College of Veterinary Medicine, Yunnan Agricultural University, No. 452 Fengyuan Road, Panlong District, Kunming 650201, China; chris_wong001@yeah.net (K.W.); 17783722977@163.com (Y.H.); wuyu295928@163.com (Y.W.); xujie2454@163.com (J.X.); zhaoyiyi913@163.com (Y.Z.)

**Keywords:** *Escherichia coli*, 4-phenylbutyrate, gut microbiota, fecal microbiota transplantation, colitis

## Abstract

Pathogenic *Escherichia coli* (*E. coli*) is a widely distributed pathogen that can cause varying degrees of zoonotic diseases, and infected animals often experience intestinal inflammation accompanied by diarrhea and dysbiosis. Previously, for the first time, we isolated *Escherichia coli* primarily of type B2 from a large-scale dairy farm in Yunnan, China. The 16s rRNA sequencing showed significant differences in the gut microbiota of calves infected with B2 *E. coli*, with higher abundance of harmful bacteria and lower abundance of beneficial bacteria compared with healthy calves. The metabolomics indicated that the concentrations of oxoadipic acid, 16-oxopalmitate, oerillyl alcohol, palmitoleic acid, and 4-phenylbutyrate (4-PBA) were significantly higher in the healthy group than in the infected group. The mouse model was established to assess the regulatory effect of 4-PBA on *E. coli*-induced colitis. Both oral administration of 4-PBA and fecal microbiota transplantation (FMT) had strong resistance to *E. coli* infection, improved survival rate and body weight, reduced intestinal tissue damage, decreased the levels of pro-inflammatory cytokines (TNF-α, IL-6, and IL-1β), and restrained TLR4/MyD88/NF-κB pathway. Our study demonstrated that 4-PBA could relieve *E. coli*-induced colitis by improving gut microbiota structure and inhibiting the expression of pro-inflammatory cytokines through the TLR4/MyD88/NF-κB pathway. The present finding reveals the therapeutic potential of the gut-microbiota-derived metabolite 4-PBA for the treatment of colitis caused by *E. coli*.

## 1. Introduction

Diarrhea in calves is mainly caused by viruses, parasites, bacteria, and nutritional and infectious factors, and it is mostly clustered and acute [1]. In the first few weeks of calf birth, due to the immature development of their digestive and immune systems, they are susceptible to external invasion leading to diarrhea. Acute infectious calf diarrhea has a high mortality rate. In production, calf diarrhea seriously affects the early growth and development as well as the later growth, reproduction, and lactation ability of calves, causing huge economic losses to breeders around the world [2]. Researchers have repeatedly isolated pathogens including but not limited to *E.coli* from diarrheal calves from various countries from 1995 [3,4]. *E. coli* has evolved stronger pathogenicity through long-term genetics and evolution. Pathogenic *E. coli* not only has more genotypes but also more and more serotypes with antibiotic resistance. In total, diarrhea caused by *E. coli* in calves is still a practical problem that needs to be solved. Some studies suggest that *E. coli* may enhance its pathogenicity in the intestines of colitis patients through specific immune escape mechanisms, thereby exacerbating the inflammatory response [5]. In some cases, the excessive proliferation of *E. coli* in the intestine can lead to an imbalance in the gut microbiota, which can exacerbate the occurrence of colitis [6].

Pathogenic *E. coli* infection typically disrupts the normal microbial composition and quantity of the gastrointestinal tract [6]. Analysis of the gut microbiome of mice infected with *E. coli* showed that the characteristics of intestinal inflammation were accompanied by an increase in bacterial load, a decrease in species diversity and bacterial migration, and a significant reduction in *bifidobacteria* and *lactobacilli*. The gut microbiota co evolves with the host gut immune system, playing an important role in regulating the expression of immune mediators and the development, recruitment, and differentiation of immune cells [7]. One way in which the gut microbiota directly inhibits diarrheal pathogens is by competing for nutrients to exclude pathogens [8], while also secreting antibacterial compounds that further inhibit the growth of harmful bacteria, such as bacteriocins or small molecule metabolites [9,10]. Therefore, the metabolites of gut microbiota are crucial for maintaining good balance in the intestine and play an important role in combating some pathogen infections. For the first time, we isolated B2-type highly pathogenic *E. coli* dominated by O3, O4, and O103 from the feces of diarrheal calves in a large-scale dairy farm in Yunnan Province [11]. These strains are resistant to gentamicin, cefazolin, chloramphenicol, ciprofloxacin, and norfloxacin and carry five resistance genes, namely, blaOXA, blaTEM, aac (6′) GIb, oqAB, and aadA1, as well as nine virulence genes, namely, fimH, Crl, iucD, astA, hlyE, ompA, ompC, K88, and papC. Currently, antibiotics are predominantly used for treating this disease, but their prolonged use promotes the development of resistant strains, disrupting the diversity of the gut microbiota [12,13]. Consequently, the development of safe and effective antibiotic alternatives or preventive measures holds significant importance.

Previous studies have shown that the combination of certain gut metabolites and antibiotics can effectively combat infections caused by pathogenic bacteria [14]. 4-Phenylbutyric acid sodium (4-PBA) is a derivative of the short-chain fatty acid butyric acid, distinguished by the presence of a phenyl group at the 4-position. It exhibits various biological activities in organisms, including anti-inflammatory, antioxidant, anti-fibrotic, and cell-stress-inhibiting effects [15]. This compound has been utilized in preclinical and clinical research for treating various diseases such as urea cycle disorders [16], spinal muscular atrophy [17], sickle cell anemia [18], and cancer [19]. Recent studies have shown that 4-PBA suppresses oxidative stress by attenuating endoplasmic reticulum stress and exerts anti-inflammatory effects by inhibiting the activity of transcription factor nuclear factor κB (NF-κB) [20]. Additionally, 4-PBA has demonstrated efficacy in treating neurodegenerative diseases like Parkinson’s disease [21]. Despite its numerous beneficial effects, limited research has explored the potential benefits of 4-PBA during infections. Initially observed in a rabbit model infected with Shigella, 4-PBA was found to inhibit the downregulation of the antimicrobial protein Catabolite Activator Protein-18 (CAP-18) mediated by Shigella [22], thereby reducing clinical disease in rabbit lung and intestinal epithelial cells. CAP protein is closely related to the virulence and colonization of *E. coli*; it participates in regulating the transcription of specific structural genes and regulating the metabolism of carbon sources [23,24]. However, the current research on the mechanism of 4-PBA against *E. coli* infection is unclear. Based on these previous studies highlighting the benefits of 4-PBA in various disease models, we aim to investigate whether 4-PBA is beneficial to the host during pathogenic *E. coli* infections. Therefore, by identifying microbial metabolites or secretions that favor the gut microbiota environment, new treatment options can be provided for *E. coli* infections. We performed 16s rRNA sequencing and metabolomics on infected and uninfected *E. coli* calves, and we screened for multiple metabolites with significant differences, including 4-PBA. By verifying the function and mechanism of action of the screened small molecule metabolite 4-PBA, its role in diarrhea and intestinal inflammation caused by *E. coli* was elucidated, providing a basis for developing new strategies for the prevention and treatment of *E. coli*.

## 2. Results

### 2.1. Healthy Calves and Diarrheal Calves Exhibited Distinct Differences in Gut Microbiota

**Figure 1:**
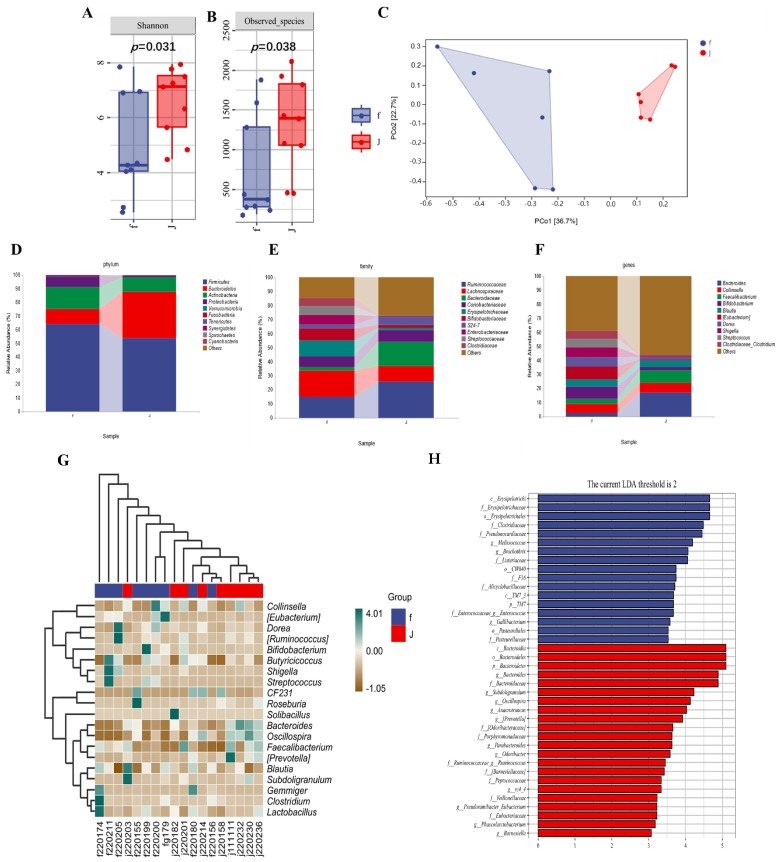
**Changes in the structure of fecal microbiota in diarrhea and healthy calves.** (**A**) Microbial community diversity was measured by the Shannon index. (**B**) Evaluating the species richness of a community using the Observed_species index. (**C**) Principal coordinate analysis (PCoA) based on the weighted UniFrac distance matrix. (**D**) Analysis of the taxonomic composition of species at the phylum level. (**E**) Analysis of the taxonomic composition of species at the family level. (**F**) Analysis of species taxonomic composition at the genus level. (**G**) Differential abundance taxonomic groups of fecal microbiota between healthy and diarrheal calves analyzed by LEfSe. LDA score ≥ 2. (**H**) Correlation heatmap of gut microbiota abundance between the healthy group and diarrhea group. The colors range from brown (negative correlation) to green (positive correlation). f represents diarrheal calves, and j represents healthy calves.

The investigation into the microbial environment within calves infected by *E. coli* was conducted through the application of microbiomics, utilizing 16S rRNA gene sequencing to analyze the fecal microbiota of both diarrheic and healthy calves. The α-diversity between the two groups was assessed using the Shannon and Observed_species indices. Analysis of the Shannon diversity index revealed that the microbial diversity in the healthy group was significantly higher than that in the diarrheic group (*p* = 0.031) (Figure 1A). Additionally, the Observed_species richness index indicated that the total microbial abundance was significantly greater in the healthy group compared to the diarrheic calves (*p* = 0.038) (Figure 1B). Furthermore, principal coordinates analysis (PCoA), principal component analysis (PCA), and non-metric multidimensional scaling (NMDS) based on weighted UniFrac distances demonstrated a dispersed distribution of data points for groups F (diarrheic) and J (healthy) on the graphs, with 95% confidence intervals for group connections largely separating the two groups, indicating significant differences in the gut microbiota between the diarrheic (F group) and healthy (J group) calves (Figure 1C). These results suggested that the richness of the microbiota is significantly higher in the healthy group compared to the diarrheic group.

The relative abundance of fecal bacteria at the phylum level revealed that Firmicutes, Bacteroidetes, Actinobacteria, and Proteobacteria dominate the fecal microbiota in both healthy and diarrheal groups, with other phyla constituting less than 1%. Diarrhea induced by *E. coli* altered the microbial composition of neonatal calf feces, increasing the abundance of Firmicutes, Actinobacteria, and Proteobacteria, while decreasing that of Bacteroidetes (Figure 1D). At the family level, Lachnospiraceae, Erysipelotrichaceae, and Bifidobacteriaceae were more abundant in the diarrhea group, with most bacteria being Gram-negative, whereas Ruminococcaceae dominated in the healthy group (Figure 1E). At the genus level, there were 10 genera that significantly differed between the diarrhea and healthy groups, notably with Bacteroides being more abundant in the healthy group (Figure 1F). Further LefSe analysis identified 39 species with significant abundance differences between groups F and J, with 17 in the diarrhea group and 22 in the healthy group. Under an Linear Discriminant Analysis (LDA) threshold greater than 2.0, Bacteroidia had the highest LDA score in the healthy group, while Erysipelotrichia scored highest in the diarrhea group (Figure 1G). As shown in Figure 1H, Solibacillus, Bacteroides, Oscillospira, Blautia, and Subdoligranum were significantly positively correlated with healthy calf groups, whereas Collinsella, Eubacteria, Shigella, Streptococcus, and Clostridium were significantly positively correlated with diarrhea calf groups. Kyoto Encyclopedia of Genes and Genomes (KEGG) enrichment pathway analysis revealed that the differentially expressed species were enriched in the biosynthesis of amino acids, fatty acids, nucleotides, and nucleosides in the biosynthetic pathway (Figure S2). Overall, the above data suggest that infection with *E. coli* may cause damage by affecting the structure of the gut microbiota.

### 2.2. Differences in Fecal Metabolites Between Diarrheal Calves and Healthy Calves

**Figure 2:**
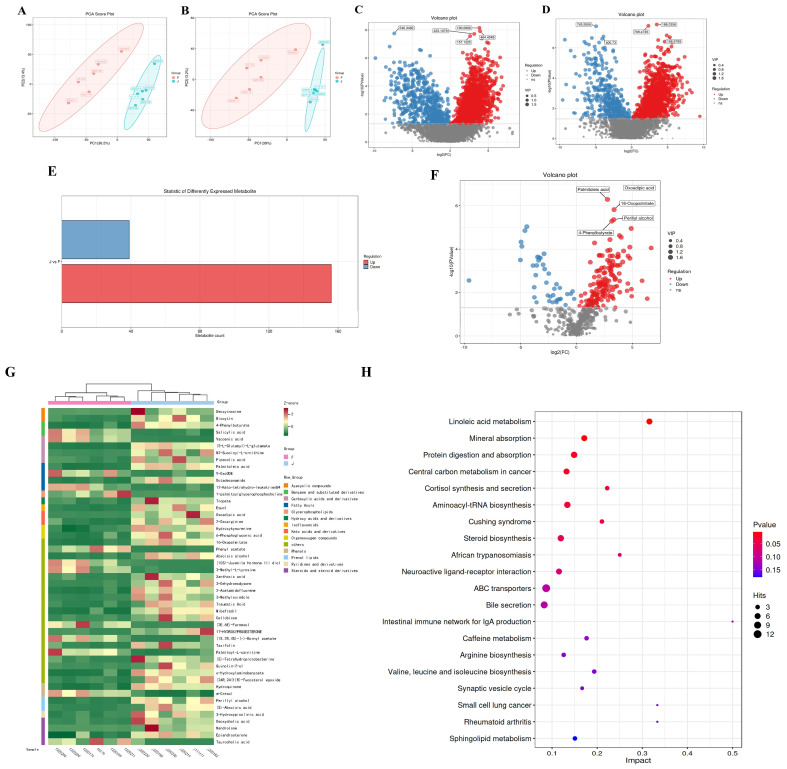
**Metabolomic changes in diarrhea and healthy calf feces.** (**A**,**B**) PCA analysis of diarrhea and healthy calf fecal samples, where A represents positive ion composition analysis and B represents negative ion composition analysis. (**C**) Volcanic diagram of differences in MS primary metabolites of positive ions. (**D**) Volcanic diagram of differences in MS primary metabolites of negative ions. (**E**) Statistical bar chart of differential metabolites under MSMS secondary analysis. (**F**) Statistical volcano plot of differential metabolites under MSMS secondary analysis. (**G**) Cluster of the relative abundance of metabolites using a UPGMA dendrogram and displaying it in the heatmap. Display of the metabolite changes using Z_Score. (**H**) Bubble diagram of the influencing factor of the KEGG metabolic pathway. f represents diarrheal calves, and j represents healthy calves.

Through metagenomic analysis, we discovered a close relationship between the metabolic pathways of gut microbiota and gut microbiome health. By conducting metabolomic analysis on fecal samples, we aimed to identify the metabolic functions of diarrheic calves. The PCA results demonstrated clear separation in both positive ion mode (R2X = 0.581) and negative ion mode (R2X = 0.522), indicating good model stability (Figure 2A,B). In the positive ion mode, we identified a total of 1880 upregulated differentially expressed metabolites (DEMs) and 654 downregulated DEMs (Figure 2C), while in the negative ion mode, we found 1730 upregulated DEMs and 426 downregulated DEMs (Figure 2D). Significantly different metabolites were selected based on *p* value and variable importance in projection (VIP), using VIP > 1 and *p* < 0.05 as criteria to filter differential metabolites between groups F and J. A total of 503 differential metabolites were identified in both positive and negative ion modes, with 156 upregulated metabolites and 39 downregulated metabolites (Figure 2E,F). The top five metabolites with the smallest *p* values, oxoadipic acid, 16-oxopalmitate, oerillyl alcohol, palmitoleic acid, and 4-phenylbutyrate, were significantly upregulated in the healthy group (*p* < 0.0001) (Figure 2F and Figure S3).

Based on UPLC-MS/MS, a non-targeted metabolomic approach was employed to screen and evaluate potential metabolites associated with *E. coli* infection. Metabolite classification revealed 50 metabolites identified as potential biomarkers (Figure 2G). Among these, benzene and its derivatives, carboxylic acids and their derivatives, and fatty acids were upregulated in the healthy group but downregulated in the diarrheal group, suggesting a potential therapeutic role of these metabolites in *E. coli*-induced diarrhea. Differential metabolites were subjected to KEGG pathway enrichment analysis using MetaboAnalyst (www.metaboanalyst.ca. Access date: 7 August 2023), with a threshold of *p*-value greater than 0.05 (pathway impact > 0.05) to select significant metabolic pathways. The results indicated enrichment of differential metabolites in pathways such as linoleic acid metabolism, mineral absorption, protein digestion and absorption, central carbon metabolism in cancer, and cortisol synthesis and secretion (Figure 2H). It implies that during intestinal microbial dysbiosis, fundamental metabolic processes of the microbiota such as protein digestion and absorption as well as fatty acid metabolism are reprogrammed, linking these metabolic pathways to energy balance and immunity, which are crucial for maintaining a healthy gut microbiota.

**Figure 3:**
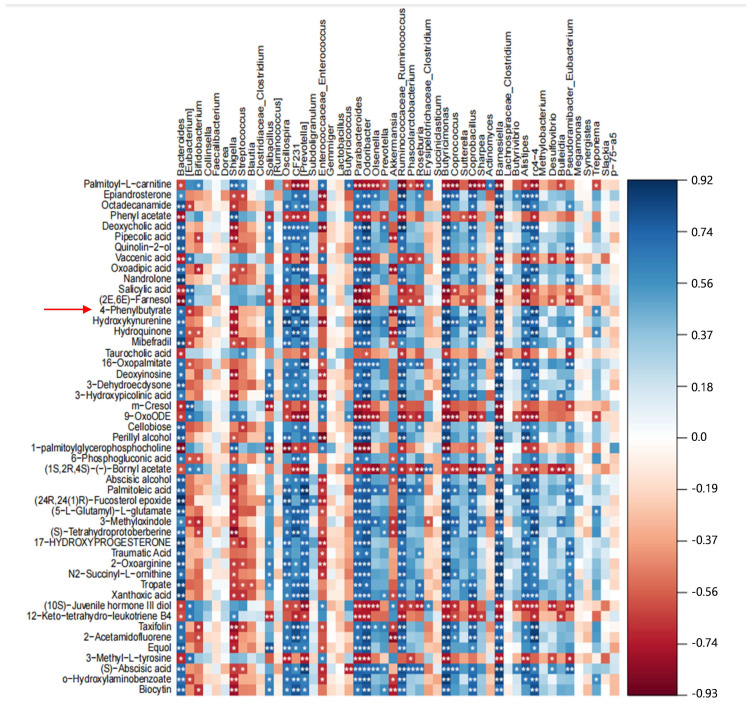
**Heat map of correlation between intestinal flora and significantly different metabolites.** The colors range from red (negative correlation) to blue (positive correlation). * *p* < 0.05, ** *p* < 0.01.

Through comprehensive analysis of 16S rRNA and metabolomics, the correlation between differential metabolites and differential microbial communities was demonstrated. The results showed that sodium 4-phenylbutyrate (Figure 3, red arrow) was positively correlated with Bacteroides, Oscillospira, Prevotella, Parabacterioids, Ruminococcaceae, Butyricimonas, Coppobacillus, Barnesiella, and Treponema. It is negatively correlated with Eubacteria, Shigella, and Akkermansia (Figure 3). Among them, Bacteroides and Prevotella belong to beneficial bacteria in the intestine, while Shigella belongs to harmful bacteria in the intestine [25]. Therefore, we have preliminarily screened a potential gut-microbiota-related metabolite 4-phenylbutyrate that may have anti-*E. coli* inflammation properties.

### 2.3. Oral Administration of 4-PBA Alleviated Inflammatory Damage Caused by E. coli in Mice

**Figure 4:**
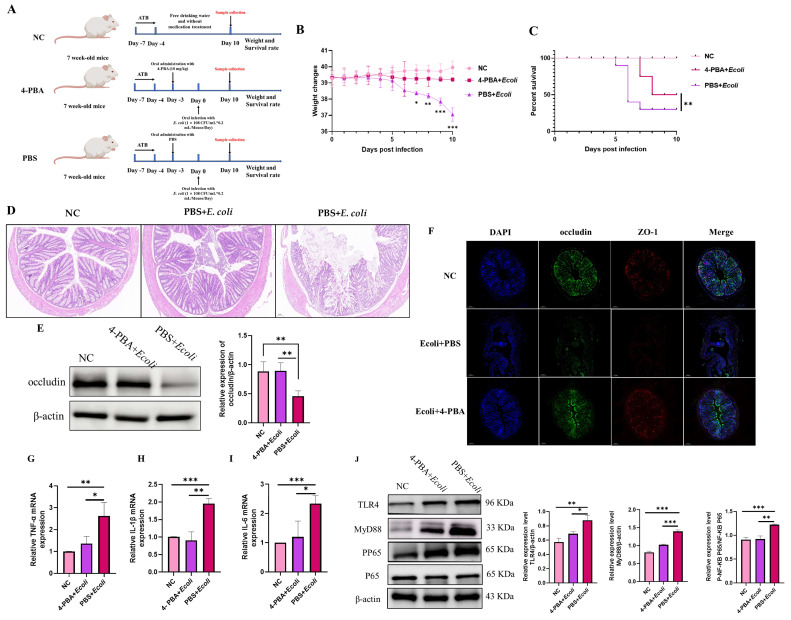
The toxicity effect of gut-microbiota-related metabolite 4-PBA on *E. coli*-induced mice. (**A**) Mouse infection model and treatment schematic diagram. (**B**) The weight changes of mice infected with *E. coli* for ten days. Statistical significance was determined using two-way ANOVA; * *p* < 0.05, ** *p* < 0.01, *** *p* < 0.001. (**C**) The survival rate changes of mice infected with *E. coli* for ten days. Statistical significance was determined using two-way ANOVA; ** *p* < 0.01. (**D**) Changes in colon tissue sections of mice infected with *E. coli* for ten days. (**E**) Protein immunoblotting for detecting the expression level of Occludin, ** *p* < 0.01. (**F**) Immunofluorescence detection of the expression levels of Occludin and ZO-1 in mouse colon tissue. (**G**–**I**) QPCR detection of mRNA levels of inflammatory cytokines in cells, where (**G**) represents for tumor necrosis factor alpha (TNF-α), (**H**) represents for interleukin-1beta (IL-1β), and (**I**) represents for interleukin-6 (IL-6). Means and SD from three independent experiments are shown; * *p* < 0.05, ** *p* < 0.01, *** *p* < 0.001. (**J**) Detection of the TLR4/MyD88/NF-κB signaling pathway by protein immunoblotting. Means and SD from three independent experiments are shown; * *p* < 0.05, ** *p* < 0.01, *** *p* < 0.001.

To investigate whether intervention with the small molecule metabolite 4-PBA can alleviate intestinal inflammation caused by *E. coli* infection, we administered 4-PBA to mice infected with *E. coli* (Figure 4A). Mouse vitality was assessed based on changes in body weight and survival rate. Results revealed that after 10 days of infection, the normal control group showed no significant activity impairment or clinical symptoms, with a 100% survival rate. In contrast, mice in the PBS + *E. coli* group exhibited a significant average weight loss and decreased survival rate within 10 days of continuous infection. The survival rate of mice in the 4-PBA + *E. coli* group was 77.78%, significantly higher than that of the PBS + *E. coli* group (Figure 4B,C). Furthermore, histological analysis of colonic tissue sections stained with H&E showed mild damage and inflammatory cells in the colons of mice pre-administered with 4-PBA compared to the NC group. In contrast, colons of mice without 4-PBA administration exhibited disrupted colonic villi structure post-*E. coli* infection, with significant infiltration of inflammatory cells, mesenteric swelling, and pronounced histological changes (Figure 4D). In addition, the tight junction protein Occludin in the colon tissue of each group of mice was examined by protein immunoblotting, and the results showed that Occludin was significantly downregulated after infection. However, there was no significant difference between the 4-PBA + *E. coli* group and the NC group, which was significantly higher than the PBS group (Figure 4E). Furthermore, ZO-1 protein and Occludin protein in mouse colon tissue slices were used for immunofluorescence detection, as shown in Figure 4F. The green fluorescence (Occludin) and red fluorescence (ZO-1) of PBS + *E. coli* were significantly lower than the other two groups. Occludin is a cell membrane protein that tightly connects the gaps between cells. ZO-1 is an important cytoskeletal protein involved in the formation and maintenance of intercellular connections [26]. These results indicated that mice pretreated with 4-PBA showed a significant reduction in intestinal damage after infection with *E. coli*. The transduction and activation of MyD88 signaling mediate host immunity and resist external invasion, but this can cause excessive production of inflammatory factors in cells, leading to inflammatory damage [27]. We were interested in whether the colitis caused by *E. coli* in mice is related to this process. Firstly, we examined the transcription levels of inflammatory factors in colon tissues, and the results showed that the mRNA accumulation levels of inflammatory factors TNF-α, IL1β, and IL6 were significantly increased in the PBS + *E. coli* group, while there was no significant difference in the levels between the 4-PBA + *E. coli* group and the healthy group (NC) (Figure 4G–I). Additionally, we examined the expression of the pathogen recognition receptor TLR4 protein and the signal adaptor protein MyD88 expression, while also detecting the downstream key inflammatory molecule NF-κB. The nuclear translocation of NF-κB p65 is a major inducer of inflammatory cytokine production [28]. The results showed that *E. coli* infection in mice activated the TLR4/MyD88/NF-κB signaling pathway, but mice pretreated with 4-PBA did not obviously activate the TLR4/MyD88/NF-κB signaling pathway (Figure 4J). The above results demonstrated that 4-PBA metabolites in the gut of mice can alleviate intestinal inflammation caused by *E. coli*, thereby reducing damage weight loss caused by *E. coli*-induced colitis. The gut microbiota co-evolves with the host gut immune system and has a significant impact on the expression of immune mediators in the gut immune response. Therefore, we used FMT to further determine whether 4-PBA inhibits intestinal inflammation caused by *E. coli* infection by affecting the gut microbiota.

### 2.4. Transplanting Fecal Microbiota from Mice Treated with 4-PBA Can Alleviate Inflammatory Damage Caused by E. coli

**Figure 5:**
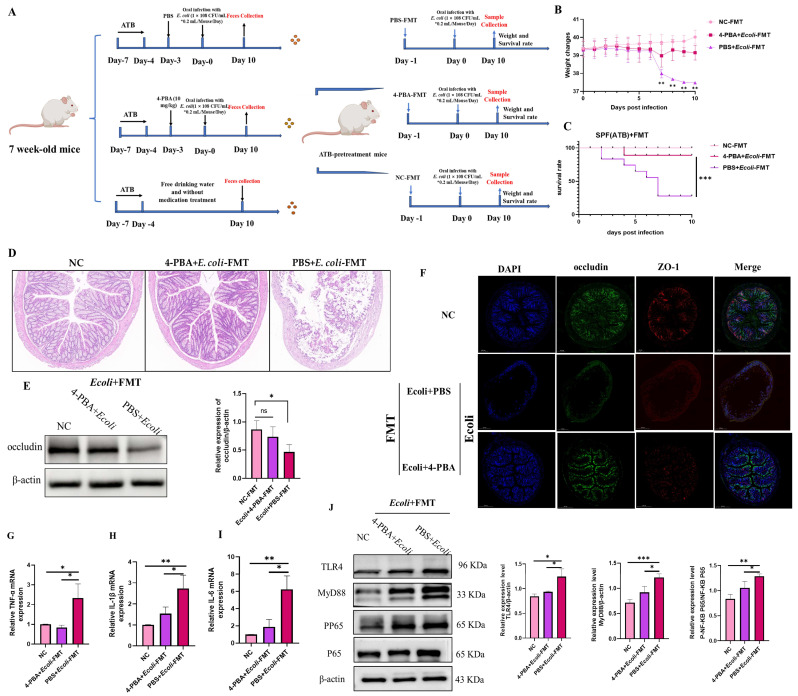
**The effect of 4-PBA pretreatment of mouse donor FMT on *E. coli* enteritis in mice**. (**A**) Illustration of FMT experiments, the * in the figure represents multiplication (**B**) The weight changes of mice infected with *E. coli* for ten days. Statistical significance was determined using two-way ANOVA; ** *p* < 0.01. (**C**) The survival rate changes of mice infected with *E. coli* for ten days. Statistical significance was determined using two-way ANOVA; *** *p* < 0.001. (**D**) Changes in colon tissue sections of mice in NC group, PBA + *E. coli* FMT group and PBS + *E. coli* FMT group. (**E**) Protein immunoblotting for detecting the expression level of Occludin, * *p* < 0.05. (**F**) Immunofluorescence detection of the expression levels of Occludin and ZO-1 in mouse colon tissue. (**G**–**I**) QPCR detection of mRNA levels of inflammatory cytokines in cells, where (**G**) represents for tumor necrosis factor alpha (TNF-α), (**H**) represents for interleukin-1beta (IL-1β), and (**I**) represents for interleukin-6 (IL-6). Means and SD from three independent experiments are shown; * *p* < 0.05, ** *p* < 0.01. (**J**) Detection of the TLR4/MyD88/NF-κB signaling pathway by protein immunoblotting. Means and SD from three independent experiments are shown; * *p* < 0.05, ** *p* < 0.01, *** *p* < 0.001.

To investigate whether the gut microbiota environment of mice treated with 4-PBA was improved and protected from damage by *E coli*, we performed FMT on mice before they were infected with *E. coli* (Figure 5A). The line graph of mouse weight and survival rate showed that after infection with *E. coli*, the fecal microbiota of mice treated with 4-PBA in advance was transplanted, and their weight and health status were restored, with no significant difference compared to the healthy group (NC). However, the fecal microbiota of mice treated with PBS transplantation showed a significant decrease in weight and survival rate, with no improvement effect (Figure 5B,C). The HE staining results of colon sections showed that compared with the healthy group and the PBS + *E. coli* FMT group, the 4-PBA + *E. coli* FMT group had slight inflammatory cell infiltration (Figure 5D) but no significant damage to tissue morphology. In addition, the protein results of Occludin showed that the tight junction protein recovered with the FMT of 4-PBA (Figure 5E) and the immunofluorescence results of ZO-1 and Occludin proteins on colon tissue also demonstrated that the FMT of 4-PBA could prevent the destruction of intercellular connections caused by *E. coli* infection (Figure 5F). The mRNA level detection of inflammatory factors showed that FMT treatment with 4-PBA reduced the release of inflammatory factors and alleviated inflammation (Figure 5G–I). Finally, in the inflammatory signaling pathway, the 4-PBA + *E. coli* FMT group showed no significant activation of TLR4/MyD88/NF-κB and was significantly reduced compared to the PBS + *E. coli* FMT group (Figure 5J). These results demonstrated that transplanting gut microbiota from mice pretreated with 4-PBA to mice infected with *E. coli* through fecal microbiota transplantation can have therapeutic and improvement effects, indicating that 4-PBA can affect gut microbiota and improve the microbiota environment.

## 3. Discussion

*E. coli* has often been isolated from animal populations with clinical outbreaks of diarrhea. According to the pathogenic symptoms, it can be divided into six types of disease prototypes: enteropathogenic *E. coli* (EPEC), enterohemorrhagic (Shiga toxin producing) *E. coli* (EHEC/STEC), enterotoxigenic *E. coli* (ETEC), enteroaggregative *E. coli* (EAEC), intestinal invasive *E. coli* (EIEC), and diffuse adhesive *E. coli* (DAEC) [29]. According to genetic typing, it can be divided into A, B1, B2, and D. Among them, the B2 strain is often considered a highly virulent strain [30]. Consistent with this, the B2 strain we isolated carries virulence and resistance genes that cause serious harm to animals. Due to its genetic characteristics, the pathogenic molecular mechanism is complex [31], and the irrational use of antibiotics increases the difficulty of prevention and treatment. Therefore, we need to develop more efficient and safe drugs or treatment methods. More and more studies have shown that traditional Chinese medicine and some small molecule compounds can have unexpected antibacterial and anti-inflammatory effects [32,33,34], and their mechanism of action is beneficial for the stability of gut microbiota. The gut microbiota and its metabolites have been shown to jointly affect life processes such as metabolism and immunity in the body [35]. Numerous studies in the past have confirmed that the occurrence of many diseases is related to the downregulation of gut microbiota [36]. This study found that calves infected with B2 pathogenic *E. coli* exhibited severe diarrhea. Compared with non-infected calves, the overall diversity of gut microbiota was significantly reduced, with the most prominent being the abundance of Bacteroidetes. In addition, the most significant increase in abundance was observed in the erysipelothrix. Previous studies have shown significant changes in gut diseases caused by Bacteroidetes and erysipelas [37,38]. Moreover, the abundance of Bacteroidetes leads to the initiation of the tricarboxylic acid cycle, utilizing glutamine and succinyl-CoA to produce δ-aminovaleric acid. The content of δ-aminovaleric acid was positively correlated with the abundance of Proteobacteria, resulting in ecological imbalance [39]. Therefore, the higher abundance of gut microbiota in healthy compared to diarrheic states is not absolute. Research by Zhang et al. [40] suggested that the decrease in the relative abundance of Bacteroidetes during diarrhea is due to an increase in the Firmicutes/Bacteroidetes ratio. These results implied that Bacteroidetes may act as probiotics protecting the host from *E. coli* infections. Consistent with previous studies, *E. coli* infections disrupt the intestinal microbiota, leading to an increase in Firmicutes and Proteobacteria abundance and a decrease in Bacteroidetes abundance [41]. This indicates that diarrhea caused by *E. coli* infection is accompanied by changes in the gut microbiota environment, which may disrupt the symbiotic balance between probiotics and harmful bacteria. The metabolomics results showed significant differences in biosynthesis and metabolism between healthy and diarrheic cattle, with variations in proteins, sugars, and fatty acids. Metabolite screening revealed upregulation of palmitoleic acid, oxoadipic acid, 16-oxopalmitate, perillyl alcohol, and 4-phenylbutyrate in the healthy group, indicating host immune responses and repair mechanisms triggered by *E. coli* infection, involving new synthetic or metabolic pathways.

In production practice, changes in factors such as environment, production, and diet can all lead to disruption of gut microbiota, making cattle susceptible to infection. Intestinal microbiota transplantation is currently applied in the treatment of various diseases, and it has been reached consensus to the occurrence and development of gastrointestinal diseases can be intervened and treated through the transplantation of specific probiotics [42]. Moreover, the biological functions of these beneficial bacteria in the intestine largely depend on their metabolites [43,44]. This finding suggests the potential for gut metabolites to regulate gut microbiota in combating infection. In anti-infection events, studies have found that 4-PBA can limit the infection of influenza A virus (IAV) by reducing unfolded protein response [45]. In addition, it inhibits bovine parainfluenza virus type 3 infection by reducing autophagy caused by endoplasmic reticulum stress [46]. However, further research is needed on the role of 4-PBA in bacterial infections. 4-PBA is known as a chemical chaperone as it acts as an inhibitor of endoplasmic reticulum stress, directly targeting mutated/unfolded proteins within cells [47]. Many studies have confirmed the role of 4-PBA in controlling inflammation primarily alleviates inflammation by reducing endoplasmic reticulum stress, including colitis [48]. However, research on 4-PBA in infectious inflammation is limited. Initially shown in a rabbit model infected with Shigella, 4-PBA inhibited the downregulation of the antimicrobial protein CAP-18 mediated by Shigella, thereby reducing clinical disease in rabbit lung and intestinal epithelial cells [20,21]. To investigate whether 4-PBA can resist *E. coli* infection or alleviate the damage caused by *E. coli* infection, we administered 4-PBA to mice in advance, and they showed significantly stronger resistance to *E. coli*. Afterwards, we transplanted fecal microbiota from mice pre administered with 4-PBA through FMT. Even after being infected with the large intestine, they were able to maintain a high level of resistance and showed significant improvement in colon health. Based on the above, the abundance of 4-PBA in animal bodies may be closely related to animal health, not only for the prevention of inflammation, but also for the defense against foreign pathogens. The results of FMT suggest that 4-PBA may be beneficial for the reproduction of some probiotics and the regeneration of 4-PBA in animal bodies, and this beneficial balance establishes a stable resistance in the animal intestine. Previous studies have shown that FMT has the potential to improve growth performance [49]. In fact, in order to further our results, 16s rRNA gene sequencing should also be performed to study the microbial composition of FMT donors and recipients, in order to compare the changes before and after FMT. Additionally, the efficacy of FMT is influenced by individual differences, and some patients may not have any response or even have the risk of transmission of other harmful microorganisms, so its effectiveness may be uncertain among different species or individuals. For our study, a larger number of mice is beneficial for better evaluating the effect of 4-PBA on *E. coli*-infected mice.

Innate immunity and acquired immunity are the main mechanisms for defending against pathogen infection. Inflammatory response, as a key mechanism of innate immune system damage to pathogens, releases a large amount of inflammatory factors and reactive oxygen species during excessive inflammation, leading to tissue damage [50,51]. Previous studies have confirmed that hemorrhagic and ulcerative colitis are common clinical manifestations of pathogenic *E. coli* infection [52]. Consistent with this, after infection with type B2 Escherichia coli, mice exhibit TLR4/MyD88/NF-κB activation and typical clinical symptoms and pathological changes of colitis. TLR4 is an important immune receptor that plays a crucial role in recognizing danger signals from pathogens and damaged cells, particularly in recognizing bacterial lipopolysaccharides (LPS). Research has found that it links the consumption of dietary nutrients with metabolic inflammation and gut microbiota, as mice with obese gut microbiota had high levels of LPS in their blood, and treatment with 4-PBA significantly improved gut microbiota dysbiosis caused by insulin resistance [53]. This discovery demonstrated that TLR4 was involved in the stability of gut microbiota and can act as a receptor for 4-PBA to regulate intestinal permeability. In addition, the activation of TLR4 is often associated with endoplasmic reticulum stress response. 4-PBA, as a regulatory factor of endoplasmic reticulum stress, can indirectly affect the activation of TLR4 by improving the endoplasmic reticulum stress response and alleviating the inflammatory response caused by it [54,55]. TLR4-mediated inflammation is usually achieved through MyD88-dependent or TRIF-dependent pathways, which activate signaling pathways such as NF-κB and MAPK, ultimately prompting cells to secrete pro-inflammatory cytokines such as tumor necrosis factor (TNF) and interleukin (IL-1, IL-6, IL-12), further enhancing the inflammatory response [56]. Studies have confirmed that 4-PBA targets TLR4 to inhibit endoplasmic reticulum stress and alleviate inflammation [57]. Similarly, by regulating MAPK and inhibiting NF-κB, 4-PBA can inhibit NF-κB to alleviate LPS-induced lung injury [58]. However, the study on the regulation of the TLR4/MyD88/NF-κ B pathway by 4-PBA is still worth supplementing. Here, we investigated the activation and transcription of TLR4/MyD88/NF-κB after *E. coli* infection and evaluated the destructive effect of 4-PBA on colitis in *E. coli*-infected mice and the impact of 4-PBA-FMT gut microbiota on *E. coli*-infected mice based on their vital signs, colon tissue integrity, and inflammatory cytokine levels. The results demonstrated that 4-PBA inhibited the activation of the TLR4/MyD88-NF-κB pathway; suppressed the activity of nuclear factor NF-κB; reduced the release of pro-inflammatory mediators IL-1β, TNF-α, and IL-6; and could attenuate gastrointestinal damage and inflammation caused by *E. coli*. This demonstrates that 4-PBA can regulate TLR4/MyD88/NF-κB-mediated *E. coli*-induced colitis. Fecal microbiota transplantation revealed that mice receiving 4-PBA transplantation exhibited a healthier intestinal environment compared to those receiving PBS transplantation in the context of *E. coli* infection. Objectively, our results supported the protective effect of 4-PBA on mice in *E. coli*-induced colitis. However, to a certain extent, the clinical application of 4-PBA may be limited by the size of the individual animal. Due to the dose used in our research, it may be necessary to develop biological tools to efficiently produce 4-PBA in the future, but this can still be used as a reference for new treatment schemes in severe infectious diseases.

**Figure 6:**
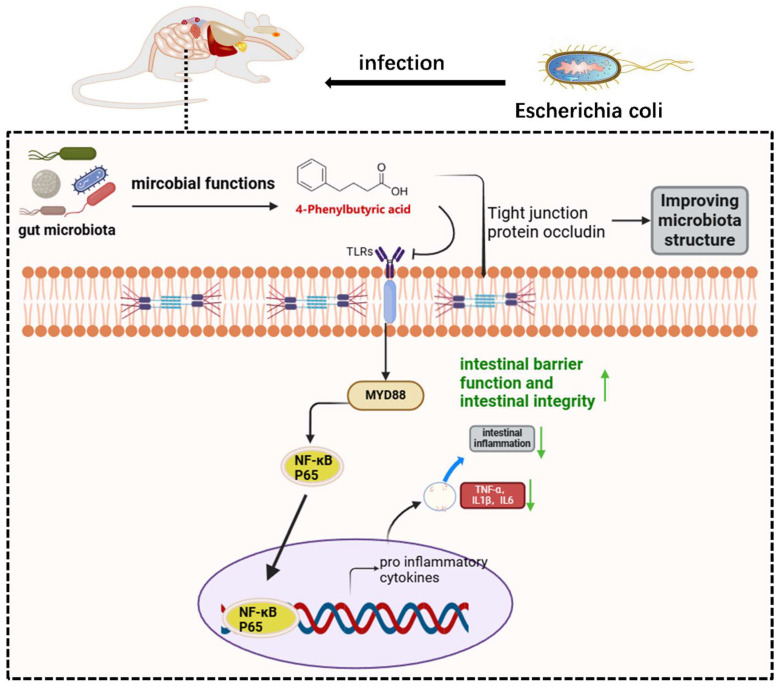
Schematic diagram of the intestinal microbiota metabolite 4-PBA inhibiting *E. coli* infection in mice.

In conclusion, our findings revealed the aberrant gastrointestinal microenvironment in calves during pathogenic *E. coli* infection, encompassing microbial abundance, composition, and metabolites. As shown in Figure 6, through the study of a mouse colitis model induced by *E. coli*, we reported the role of 4-PBA in maintaining and restoring intestinal microbiota homeostasis, which is expected to become a substitute for intervening in *E. coli* infection.

## 4. Materials and Methods

### 4.1. Bacteria, Samples, and Experimental Animals

The pathogenic *E. coli* used in this study was a B2-type pathogenic strain isolated from the feces of calves in a large-scale breeding farm in Xundian Hui and Yi Autonomous County (please refer to Figure S1 and Tables S1 and S2). The feces of 6 calves with diarrhea and 6 healthy calves were collected from the Beijing Shounong Group Yunnan Branch for microbiome and transcriptome sequencing. Specific-pathogen-free (SPF) male Kunming mice (7 weeks old) were purchased from the Animal Experimental Center of Kunming Medical University.

### 4.2. Mouse Infection and Sample Collection

The experimental content of this section is based on the experimental plan executed by our previous research institute as a reference [59]. After one week of normal feeding, SPF mice were randomly divided into three groups and pretreated with antibiotic solution (ATB). Each group of mice was given sterile water containing ampicillin (1 mg/mL), streptomycin (5 mg/mL), vancomycin (0.25 mg/mL), and colistin (1 mg/mL) for 3 days [60]. The treatment groups were as follows: NC group mice (*n* = 9) drank water freely every day and were gavaged an equal amount of PBS; PBS + *E. coli* group (*n* = 9): by oral of 1 × 10^8^ CFU/mL *E. coli* solution to simulate a mouse colitis model (1 × 10^8^ CFU/0.2 mL/mouse/day); 4-PBA + *E. coli* group (*n* = 9): oral administration of 10 mg/kg [61] of 4-PBA (Sigma Aldrich, St. Louis, MI, USA) for 3 days in advance before infection with *E. coli*. From day 0 to day 10, we observed and recorded the weight of mice and evaluated their survival rate daily throughout the entire experimental process. On the 10th day after infection, feces was processed as previously described [62], and fresh fecal samples were collected and homogenized in a sterile environment within 2 h. The samples were filtered through a 0.25 mm stainless steel cell filter and then centrifuged at 6000× *g* for 15 min to remove impurities and particles. After filtering out impurities, the sample was quickly transferred to −80 °C for storage. After the last administration, all animals were fasted and euthanized without water. The complete segmented colon tissue was collected and stored in a −80 °C freezer for later use. The distal colon tissue was fixed with 4% paraformaldehyde and used for histopathological analysis. Six mice were selected from each of the three experimental groups for weight analysis.

To investigate the correlation between gut microbiota disruption and *E. coli* infection, SPF mice were randomly assigned to three groups. Prior to fecal microbiota transplantation (FMT), the mice were pre-treated with an antibiotic solution (ATB). Fecal samples were collected one day before FMT, following the previously outlined procedure. The first group served as the negative control (NC), consisting of nine mice maintained under standard feeding and care conditions to represent a healthy control group. In the 4-PBA + *E. coli* FMT group, fecal samples from mice in this group were diluted with PBS, centrifuged at a low speed to obtain the supernatant, and then transferred to antibiotic-pretreated SPF mice (*n* = 9) before *E. coli* infection. Similarly, in the PBS + *E. coli* FMT group, fecal samples from mice in this group were diluted with PBS, centrifuged, and administered to antibiotic-pretreated SPF mice (*n* = 9) before *E. coli* infection. Six mice were randomly selected from each group for weight and survival analysis. On the 10th day post-infection, colon tissue samples were collected from the mice for the detection of post-inflammatory indices.

### 4.3. DNA Extraction and 16s rRNA Genome Sequencing

The fecal Genomic DNA Extraction Kit (TianGen, DP328, Beijing, China) was used to extract bacterial genomic DNA from 100 mg fecal samples, and a spectrophotometer and nucleic acid gel electrophoresis were used to check the concentration and purity. Using 338F and 806R as universal primers (338 F: 5′-ACT CCTACGGGAGGCAGCAG-3′, 806 R: 5′-GGA CTACHVGGGTWTCTAAT-3′), an index sequence and a linker sequence were added to the 5′ end of 338F and 806R for the design of specific primers. PCR amplification was performed on the variable region of bacterial 16S rRNA gene V3-V4 to obtain an amplification fragment of about 500 bp. The amplified DNA was purified by Novozymes VAHTSTM DNA VAHTS DNA (Vazyme, Wuhan, China), and the PCR product was quantified using a Quant iT Picogreen dsDNA quantification kit. TruSeq Nano DNA LT (Illumina, San Diego, CA, USA) was used. A PCR product library was prepared by using a library to prepare reagent kits and performed double-end sequencing on the Illumina MiSeq platform. The samples were preliminarily screened based on the sequence quality of the original high-throughput sequencing data, and those with issues were retested. Through high-quality screening, the library and samples were divided based on the index and barcode information of the original sequence, and then barcode sequences were removed.

### 4.4. Sequencing Data Analysis

The QIIME2 was used to process sequencing data [63], version 2020.02. The DADA2 method QIIME2 (April 2019) was used for primer removal, quality filtering, denoising, splicing and de chimerism, splicing and de chimerism, and other steps to obtain valid data (clean data).This method was also employed for quality control to generate amplicon sequence variants (ASVs) from each duplicate sequence. ASVs offer single-nucleotide resolution and accurately represent exact sequence variants, providing higher resolution than traditional OTU (operational taxonomic unit) clustering [64]. The RDP FrameBot software (version 1.2.0; https://github.com/rdpstaff/Framebot, accessed on 5 April 2022) was used to correct insertion and deletion errors in nucleic acid sequences. After analysis by FrameBot, corrected nucleic acid sequences and protein sequences can be obtained, which will be used for subsequent analysis. After obtaining ASV feature sequences or OTU representative sequences, we can statistically analyze their length distribution to check whether the length of these sequences is comparable to the length range of the sequencing target fragment, as well as whether there are sequences with abnormal lengths. We adopted the QIIME2 classification sklarn algorithm (https://github.com/QIIME2/q2-feature-classifier, accessed on 12 April 2022) for each ASV feature sequence or representative sequence of each OTU. Default parameters in QIIME2 software (version 2022.2) were used, and the species were annotated by using naïve Bayes. The qiime feature table rare function was used, and the flattening depth to 95% of the minimum sample sequence size was set. The ‘qiime taxa barplot’ command is called to plot. Alpha diversity indices were calculated to assess the within-sample microbial diversity, including richness and evenness metrics, based on the ASV abundance table, with parameters of “--p-steps 10--p-min depth 10--p-iterations 10”, which means the minimum flattening depth is 10, and parameter “--p-max-95%”. We used PERMANOVA (version 2.15.3, vegan package) in R software (version 4.4.2) to calculate Jaccard, Bray–Curtis, unweighted UniFrac, and weighted UniFrac for Beta diversity analysis and to evaluate intergroup differences. Linear discriminant analysis (LDA) (LEfSe, https://huttenhower.sph.harvard.edu/lefse accessed on 10 August 2024) using default parameters (LDA > 2, *p* < 0.05) was used to perform linear discriminant analysis on microbial communities or species with significant differences between different groups. R software is used for Spearman correlation analysis, analyzing the correlation between differential bacterial genera and phenotype data, as well as gut microbiota data. The annotation of microbial gene information corresponds to the functional information of the entire prokaryotic genome in KEGG data.

### 4.5. Untargeted Metabolomics and Analysis

The untargeted metabolomics analysis was conducted as previously described [32]. We slowly thawed the frozen fecal samples under ice bath conditions. A total of 100 mg of fecal samples from each individual were taken, we added precooled methanol solution containing internal standards to extract metabolites, and we vortexed the mixture for 30 s; the sample was then grinded with steel balls for 2–3 min, and after homogenization, the sample was centrifuged at 12,000 rpm at 4 °C for 10 min, and the supernatant was filtered through a 0.22 μm membrane for LC-MS detection. Based on the ultra-high efficiency liquid phase system Thermo Vanquish (Thermo Fisher Scientific, New York, NY, USA), an ACQUITY UPLC^®^ HSS T3 (2.1 × 100 mm, 1.8 µm) (Waters, Milford, MA, USA) chromatographic column was used to separate fecal samples. The column temperature was maintained at 40 °C, the flow rate was 0.3 mL/min, and the injection volume was 2 μL each time. The collection of primary and secondary spectra of fecal samples was performed using a Thermo Q Exactive mass spectrometer (Thermo Fisher Scientific, New York, NY, USA). The ionization source parameters of electric spray were positive ion spray voltage of 3.50 kV, negative ion spray voltage of −2.50 kV, sheath gas of 40 arb, and auxiliary gas of 10 arb. The capillary temperature was 325 °C, the first stage full scanning resolution was 70,000, the scanning range was *m*/*z* 100~1000, HCD was used for secondary fragmentation, the collision energy was 30 eV, the second stage resolution was 17,500, and the top 10 ions of the collected signal were fragmented. At the same time, dynamic exclusion was used to remove unnecessary MS/MS information. The Proteowizard software package (v3.0.8789) was used to process the raw data files generated by UPLC-MS/MS and perform peak integration and quantification for each metabolite. We eliminated systematic errors based on QC samples and filtered out substances with RSD > 30% in QC samples during quality control and quality assurance processes for subsequent data analysis. Statistical analysis was conducted using the R software package (version 4.4.2) Ropls platform, including principal component analysis (PCA), partial least squares discriminant analysis (PLS-DA), orthogonal partial least squares discriminant analysis (OPLS-DA), dimensionality reduction analysis, and single-factor analysis. Multivariate statistical analysis such as PCA, PLS-DA, and univariate statistical analysis (Student’s *t*-test, Mann–Whitney Wilcoxon U-test, ANOVA, and correlation analysis) were used. The uniform calculation method adopts the statistical analysis package widely used in R studio (http://cran.r-Project.org/, accessed on 5 April 2022). Spearman correlation analysis was used to analyze the correlation between microbial communities and metabolites. By calculating Spearman correlation coefficients to demonstrate the correlation between differential metabolites and differential bacterial populations, metabolite information annotation KEGG pathway metabolite pathway analysis was performed. If both the microbiota and metabolites were annotated onto the same KEGG pathway, it means they are closely related and can be analyzed for correlation.

### 4.6. Histopathological Analysis

Obtaining tissue samples through surgery or autopsy, we placed the tissue sample in a fixative (usually 10% formalin) immediately after collection. Gradually, we removed water from the fixed tissue by passing it through increasing concentrations of ethanol (typically 70%, 95%, and 100%). We replaced ethanol with a clearing agent (like xylene) to remove the alcohol and make the tissue transparent. We then immersed the cleared tissue in molten paraffin wax. Once the tissue was fully infiltrated, it was allowed to solidify in a mold, forming paraffin blocks containing the tissue sample. We used a microtome to cut thin sections (usually 4–5 µm thick) of the embedded tissue block. We then placed the tissue sections onto glass slides. The sections were typically floated on warm water to remove wrinkles before being transferred onto slides. We allowed the slides to dry for 30 min to an hour at room temperature or at 37 °C in an incubator. Hematoxylin and eosin (H&E) staining was used to distinguish cellular components, while immunohistochemistry (IHC) was used to detect the specific proteins Occludin and ZO-1. We examined the overall characteristics of tissues under a microscope, such as tissue structure, inflammatory changes, necrosis, or abnormal growth patterns. We looked for abnormal structures such as granulomas, foreign bodies, tumors, and hyperplasia. We evaluated the health status by observing the types and quantities of cells, such as nuclear atypia, mitotic activity, and cellular infiltration. We then quantitatively evaluated the number of cells to quantify the degree of inflammation, fibrosis, or tumor involvement. We associated the discovered changes with clinical and radiological data (if available). Quality control and troubleshooting: We ensured tissue fixation, ensured slice thickness and uniformity met quality requirements, and ensured consistent and interpretable staining. After the analysis was completed, we stored the slices in a safe and orderly manner.

### 4.7. Slice Immunofluorescence

After establishing the infection model, mouse colon tissue was collected and fixed with 4% paraformaldehyde, and each colon biopsy sample was embedded in paraffin to make 4 μm sections. The paraffin sections were deparaffinized to water, repaired with EDTA buffer (pH = 8.0), and washed three times with PBS. Paraffin sections were blocked in 10% BSA at 37 °C for 30 min, and the blocking buffer was removed. The paraffin sections were incubated overnight with anti-MyD88 primary antibody (1:100, Servicebio, Wuhan, China) at 4 °C. Subsequently, the slices were washed three times with PBS and incubated with a secondary antibody (1:100, Servicebio, Wuhan, China) at room temperature for 50 min. DAPI was used for incubation at room temperature in the dark for 10 min to counterstain the cell nucleus. Finally, the paraffin sections were sealed with fluorescent fixative for further analysis. After staining, fluorescence intensity was observed using a fluorescence microscope combined with the MicroPublisher imaging software (version 5.0) platform (Q-imaging).

### 4.8. Western Blotting

RIPA protein lysis buffer (Solarbio, R0010, Beijing China) was used to lyse the colonic tissue, and protein was obtained by grinding and centrifugation. The concentration was measured and normalized using a BCA protein quantification kit (Shanghai Yaase Biotechnology, ZJ102, Shanghai, China). The obtained proteins were mixed in proportion to the sample buffer, and then we performed SDS-PAGE electrophoresis and transferred them to the PVDF membrane (Merck Millipore, IPVH00010, Burlington, MA, USA). Then, the primary antibody was sealed and incubated overnight at 4 °C before reacting with the secondary antibody conjugated with horseradish peroxidase (HRP) at room temperature for visualization. We visualized the protein bands using an enhanced chemiluminescence (ECL) system (Cytiva, ImageQuant 800, Shanghai, China). The following antibodies were used: TLR4 (BIOSS, bs-2059, Beijing, China), MyD88 (proteintech, 23230-1-AP, Wuhan, China), p65 (A22684, abclonal, Wuhan, China), pp65 (abclonal, AP1294, Wuhan, China), β-actin (4967, cst, Boston, MA, USA), goat anti-mouse (BIOSS, bs-0296G-HRP, Beijing, China), and goat anti-rabbit (BIOSS, bs-80295G-HRP, Beijing, China).

### 4.9. Extraction of Total RNA and Real-Time Quantitative Reverse Transcription PCR (RT-qPCR)

RNAiso Plus reagent (takara, 9108Q, Osaka, Japan) was used to lyse the sample, and we extracted total RNA using chloroform, isopropanol, anhydrous ethanol, and other reagents according to the manufacturer’s agreement. A TransScript^®^IV kit (Transgene, AT311) was used for reverse transcription. The qTOWER3G real-time fluorescence quantitative PCR system and SYBR Green qPCR Master Mix (Universal) (MCE, HY-K0501A, Shanghai, China) were used for qRT PCR reaction. All measurements were conducted in triplicate, and the arithmetic mean of Ct values was used for calculation: the average Ct value of the target gene was standardized to its respective housekeeping gene (β-actin), the average Ct value (internal reference gene, Ct), and then to the experimental control. By using a relative quantitative method, the obtained value was taken as a power of 2-Δ Δ Ct and expressed as an n-fold adjustment compared to the experimental control 2- Δ Ct, measured in triplicate using biological methods. Used for qPCR amplification of cDNA after reverse transcription, the amplification system is shown in Tables S3 and S4. The primers for PCR are listed in Table 1.

### 4.10. Data Statistical Analysis

The data for this study were collected in triplicate and analyzed and plotted using GraphPad Prism 9 software (GraphPad Software Inc., La Jolla, CA, USA). All data were presented as mean ± standard deviation (SEM). The significant differences between groups were determined using Student’s *t*-test, and the cumulative mortality rate was calculated using the Kaplan–Meier curve (also known as survival curve) to estimate the survival rate of mice and plot the survival curve. A Mantel Cox log rank test was used for analysis, and two-way ANOVA was used for weight changes. *p* < 0.05 indicates statistical difference, *p* < 0.01 indicates significant statistical difference, and *p* < 0.001 indicates extremely significant statistical difference.

## Figures and Tables

**Table 1:** Sequences of primers used for assessing the gene expression of IL-1β, IL-6, and TNF-α.

Gene	Sequence	Product Length/bp
β-actin	Forward: CATCGTCCACCGCAAAT	103
	Reverse: GCCATGCCAATCTCATCTC	
IL-1	Forward: TGCCACCTTTTGACAGTGATG	138
	Reverse:TGATGTGCTGCTGCGAGATT	
IL-6	Forward: GCCTTCACTCCATTCGCTGTCTC	144
	Reverse: AAGTAGTCTGCCTGGGGTGGTG	
TNF-α	Forward: GCTGACGGGCTTTACCTCATCTAC	145
	Reverse: GGCTCTTGATGGCAGACAGGATG	

## Data Availability

16S rRNA gene data have been presented in the National Center for Biotechnology Information (NCBI) Sequence Read Archive (SRA) database under accession number PRJNA1158667. Metabolomics raw data have been deposited in Mendeley Data (doi: 10.17632/6crjmyww6r.1, access on 10 August 2024; https://data.mendeley.com/preview/6crjmyww6r?a=d2a0d84a-633e-4386-a926-2eea3b3cd104, accessed on 18 September 2024).

## Supplementary Materials

The following supporting information can be downloaded at https://www.mdpi.com/article/10.3390/ijms26051974/s1.
