# Supplementary material for: The Therapeutic Potential of Gut-Microbiota-Derived Metabolite 4-Phenylbutyric Acid in Escherichia coli-Induced Colitis
Source: Int J Mol Sci. 2025 Feb 25;26(5):1974. doi: 10.3390/ijms26051974 (PMC11901052; doi:10.3390/ijms26051974)
Supplement: Supplementary file 1 [file ijms-26-01974-s001.zip › ijms-3400982-supplementary.pdf]

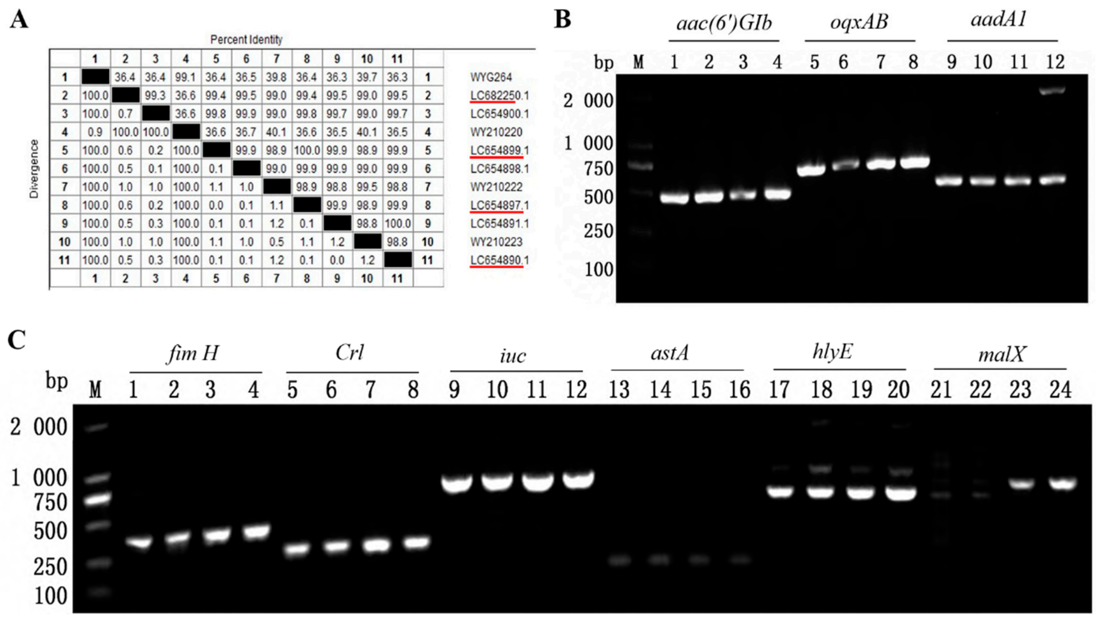

Figure S1 Biological characteristics analysis of four strains of Escherichia coli. **(A)** Homology alignment of gene sequences; **(B)** PCR detection results of antibiotic resistance genes in isolated strains; **(C)** PCR detection results of virulence genes in isolated bacterial strains

Table S1 Serotype identification of Escherichia coli

Antigen	O3	O4	O103	O6
210220		+		
210222	+			
210223			+	
G264				

Table S2 Drug sensitivity analysis and resistance gene detection results of isolated bacterial strains

Strain	Drug Resistance	Drug Sensitivity	Resistant Gene
210220	GEN、CZ、CPL、CIP、NOR	*AK*	*blaOXA*、*blaTEM*、*aac(6')GIb*、*oqxAB*、*aadA1*

210222	GEN、CZ、CPL、CIP、NOR	*AK*	*blaOXA*、*blaTEM*、*aac(6')GIb*、*oqxAB*、*aadA1*
210223	GEN、CZ、CPL、CIP、NOR	*AK*	*blaOXA*、*blaTEM*、*aac(6')GIb*、*oqxAB*、*aadA1*
G264	GEN、CZ、CPL、CIP、NOR	*AK*	*blaOXA*、*blaTEM*、*aac(6')GIb*、*oqxAB*、*aadA1*

Table S3 Real-time fluorescence quantitative reaction system

component	Volume/μL
2×TransStart®Top Green qPCR Super Mix	10
PCR Forward Primer（10 μM）	0.5
PCR Reverse Primer（10 μM）	0.5
cDNA	2.0
ddH_2O	7
Total	20

Table S4 Real-time fluorescence quantitative reaction program

Loop steps	temperature	Time	Cycle time
Pre-denaturation	94 °C	30 S	1
Denaturation	94 °C	30 S	45
Anneal/Extend	60 °C	30 S	
Melting curve	Default		1

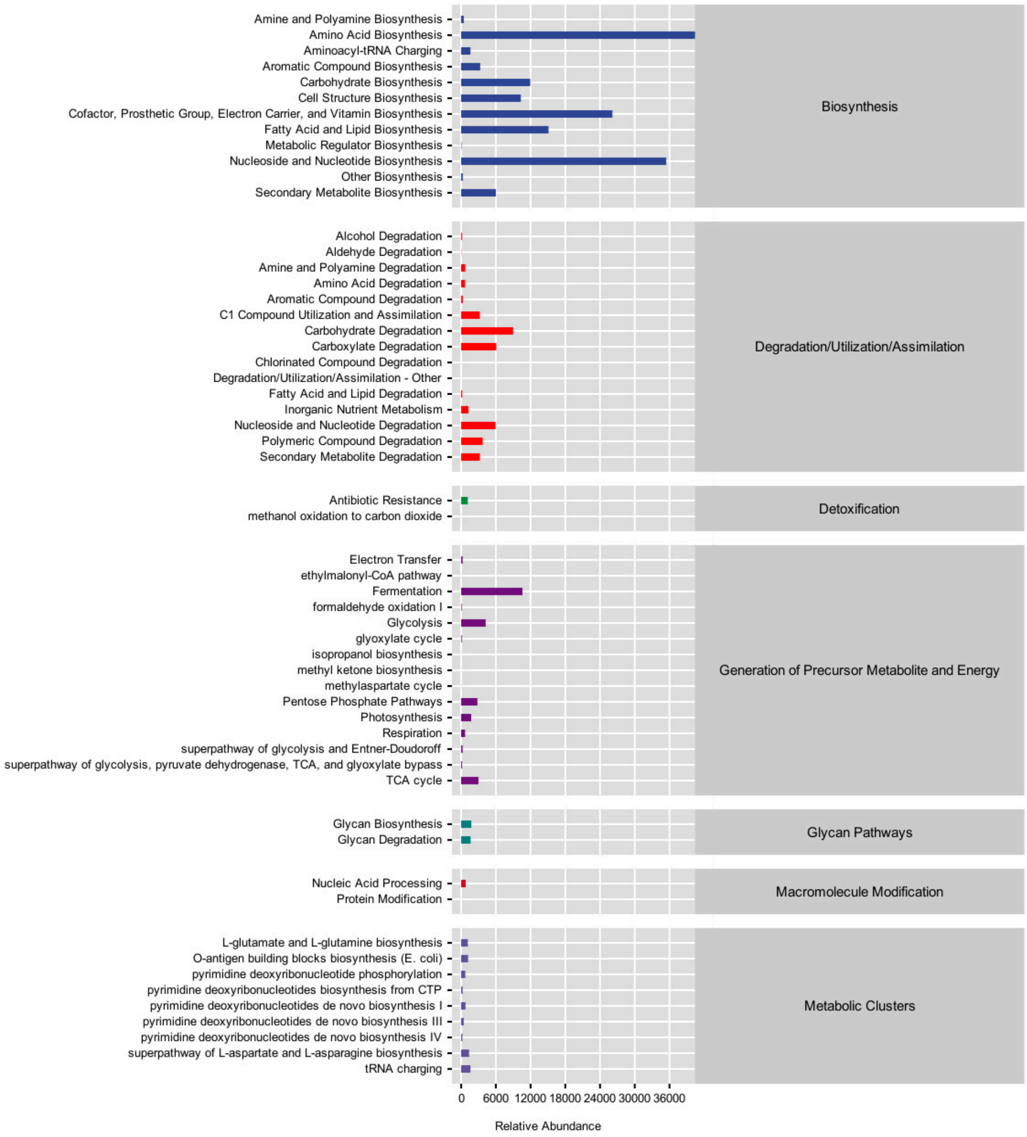

Figure S2. KEGG Enrichment Pathway Diagram.

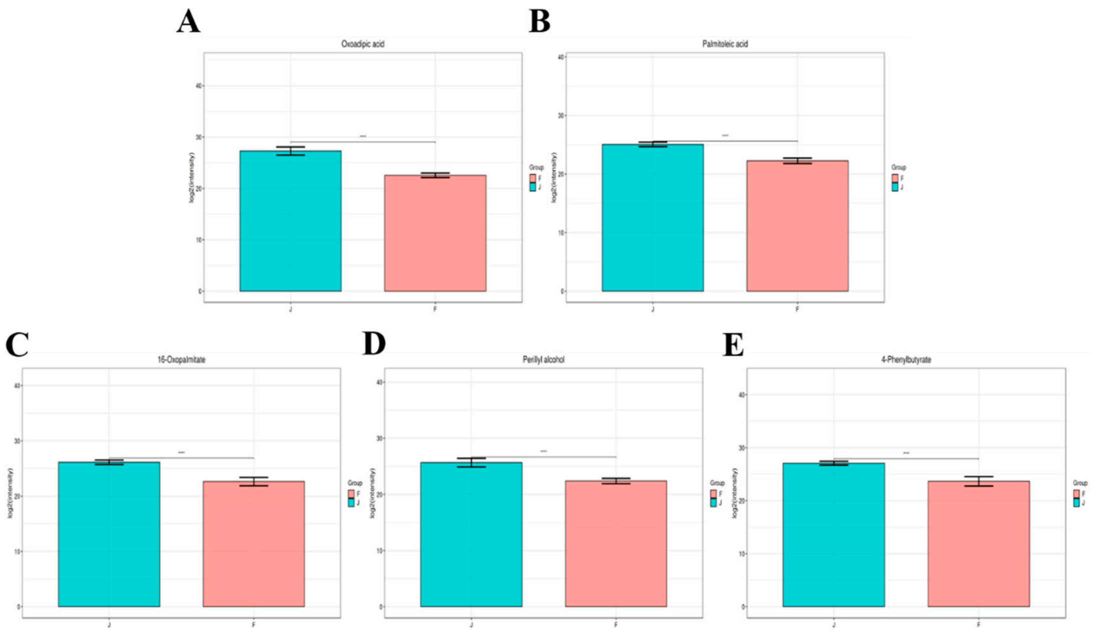

Figure S3. The top 5 metabolites with the smallest P values. **A** represents Oxoadipic acid, **B** represents Palmitoleic acid, **C** represents 16-oxopalmitate, **D** represents Oerillyl alcohol, and **E** represents 4-phenylbutyrate.
